# Immunotherapy – 2077. Allergen specific immunotherapy in allergic conjuntivitis due to dust mite

**DOI:** 10.1186/1939-4551-6-S1-P159

**Published:** 2013-04-23

**Authors:** Sanjay Pawar

**Affiliations:** 1 Allergy, Shriratna Hospital Karad, Karad, India

## Background

The safety and the efficacy of allergen specific Immunotherapy by subcutaneous route in perennial conjunctivitis caused by house dust mite were evaluated in 89 patients for 24 months.

## Methods

Group of 89 patients diagnosed by ophthalmologist included in study. Patients of allergic conjunctivitis included in this study underwent Skin allergy test and specific IgE tests for dust mite allergens. Out of 89 patients 43 received Allergen specific Immunotherapy (SIT) subcutaneous injections with standardized Dermatophgoides farinae (D.f.) and Dermatophagoides pteronyssinus (D.pt.) allergen extract. While 46 patients are only treated by conventional treatment by using local steroids and antihistaminic drops. The vaccine was supplied by a commercial company as per the prescription for each patient. The subjects generally received three concentrations of antigens ending with the most concentrated solution. Later on, maintenance immunotherapy was continued with the 1:50 concentration for 24 months. Results were evaluated on 2, 6, 12, 18 and 24 months by using Total Symptom Score (TSS), Local Medication use, Clinical examination grading, Quality of Life evaluation (QOL).

## Results

Of the 89 patients included, only 81 completed the study (43 in the Immunotherapy group and 46 in the Non Immunotherapy group). Three out of 43 (6.9%) patients dropped out because of insufficient efficacy in the Immunotherapy group compared to five out of 46 (10.8%) in the non immunotherapy group. Sum of all four primary end point criterias are scored against five group of efficacy evalution. No improvement 8 (9.8% ), marginal improvement 16 (19.7%), improvement 23 (28.3%) and significant improvement 35 (43.2%). In group of 35 improved patients 26 (74.2%) were received immunotherapy while 9 (25.7%) patients did not.

## Conclusions

Two years treatment with subcutaneous house dust mite immunotherapy significantly reduced symptoms and medication use in allergic conjunctivitis patients. This was associated with a greater subjective improvement and quality of life in patients of perennial conjunctivitis caused by house dust mites.

